# Alternative approaches to identify core bacteria in *Fucus distichus* microbiome and assess their distribution and host-specificity

**DOI:** 10.1186/s40793-022-00451-z

**Published:** 2022-11-16

**Authors:** Jungsoo Park, Katherine Davis, Geneviève Lajoie, Laura Wegener Parfrey

**Affiliations:** 1 Department of Botany, Biodiversity Research Centre, University of British Columbia, Vancouver, BC Canada; 2 Department of Zoology, University of British Columbia, Vancouver, BC Canada; 3 Institut de Recherche en Biologie Végétale, Département de Sciences Biologiques, Université de Montréal, Montréal, QC Canada

**Keywords:** Core microbiome, Macroalgal microbiome, Microbial ecology, Symbiosis

## Abstract

**Background:**

Identifying meaningful ecological associations between host and components of the microbiome is challenging. This is especially true for hosts such as marine macroalgae where the taxonomic composition of the microbiome is highly diverse and variable in space and time. Identifying core taxa is one way forward but there are many methods and thresholds in use. This study leverages a large dataset of microbial communities associated with the widespread brown macroalga, *Fucus distichus,* across sites and years on one island in British Columbia, Canada. We compare three different methodological approaches to identify core taxa at the amplicon sequence variant (ASV) level from this dataset: (1) frequency analysis of taxa on *F. distichus* performed over the whole dataset, (2) indicator species analysis (IndVal) over the whole dataset that identifies frequent taxa that are enriched on *F. distichus* in comparison to the local environment, and (3) a two-step IndVal method that identifies taxa that are consistently enriched on *F. distichus* across sites and time points. We then investigated a *F. distichus* time-series dataset to see if those core taxa are seasonally consistent on another remote island in British Columbia, Canada. We then evaluate host-specificity of the identified *F. distichus* core ASVs using comparative data from 32 other macroalgal species sampled at one of the sites.

**Results:**

We show that a handful of core ASVs are consistently identified by both frequency analysis and IndVal approaches with alternative definitions, although no ASVs were always present on *F. distichus* and IndVal identified a diverse array of *F. distichus* indicator taxa across sites on Calvert Island in multiple years. Frequency analysis captured a broader suit of taxa, while IndVal was better at identifying host-specific microbes. Finally, two-step IndVal identified hundreds of indicator ASVs for particular sites/timepoints but only 12 that were indicators in a majority (> 6 out of 11) of sites/timepoints. Ten of these ASVs were also indicators on Quadra Island, 250 km away. Many *F. distichus*-core ASVs are generally found on multiple macroalgal species, while a few ASVs are highly specific to *F. distichus.*

**Conclusions:**

Different methodological approaches with variable set thresholds influence core identification, but a handful of core taxa are apparently identifiable as they are widespread and temporally associated with *F. distichus* and enriched in comparison to the environment. Moreover, we show that many of these core ASVs of *F. distichus* are found on multiple macroalgal hosts, indicating that most occupy a macroalgal generalist niche rather than forming highly specialized associations with *F. distichus*. Further studies should test whether macroalgal generalists or specialists are more likely to engage in biologically important exchanges with host.

**Supplementary Information:**

The online version contains supplementary material available at 10.1186/s40793-022-00451-z.

## Background

There is an increasing recognition that bacteria closely associated with hosts play important roles in host development, survival, and fitness [1, 2]. With the advent of high-throughput sequencing techniques, the interest in understanding ecological and evolutionary relationships with bacteria in a variety of host systems has been explosive in multiple contexts, including epidemiology, species conservation given climate change, and microbial manipulation to improve crop yields [3–6]. The goal is often to identifying the functionally important taxa so that they can be monitored or manipulated. This is straightforward in obligate symbioses where one or a small number of symbionts and their host are consistently engaging with each other, such as for bobtail squid and *Allivibrio fischeri* [7, 8] and for siboglinid tube worms and endosymbiotic bacteria [9]. However, other hosts have a much greater degree of variability in their microbial associates; the microbiota associated with plants and macroalgae is generally comprised of hundreds to thousands of species with spatial and temporal variation across environmental gradients [10–13]. The complex nature of the microbiota in these systems complicates the task of identifying meaningful relationships between host and microbe despite the large amount of microbiome data being generated for them.

In variable host-microbe systems, a core microbiome approach might be valuable to generate hypotheses about which bacterial taxa are functionally important for host biology [14, 15]. A core microbiome generally refers to a set of microbes consistently associated with a given host [15]. Their consistent presence, often in high abundance, is thought to be a product of evolutionary and ecological processes that govern host-microbe interactions [15]. Recent studies suggest that core taxa can predict an animal’s health/disease status [16] and be used as targets for manipulation to improve crop yields or resilience in sustainable agroecosystems [17]. The rationale for spotlighting a small number of microbial taxa that are the most likely to be functionally important symbionts is clear [18–20], but there are many and varied approaches for identifying the core. Across studies, core taxa have been identified spatially [21–23] and/or temporally [24] as those above some arbitrary frequency threshold. These thresholds vary across studies, from 50% frequency in seagrasses [25] to 80% in corals [26] to 90% in amphibians [27] reflecting both the differences in the microbiota distribution and fidelity across hosts and author preferences. Another approach is to require microbes to be specifically associated with a host, defined as enriched compared to the background environment, in addition to high frequency to qualify as core [28], or to require high abundance [29]. Such inconsistent methods and flexible parameters have called into question the robustness of a core microbiome approach [30]. Taking a more conservative approach to defining the core by considering enrichment compared to the environment and/or using broad sampling across sites and over time, will likely improve the utility of the core microbiota approach, particularly for host systems with highly variable microbiomes.

The epiphytic bacteria on macroalgae, and surface bacterial communities in general, tend to be heavily influenced by environmental conditions [31, 32] and often composed of functionally redundant taxa [33]. Yet, within this variability a suite of taxa frequently found on macroalgal surfaces is emerging across studies, including Saprospiraceae, *Granulosicoccus*, and Flavobacteria [34–36]. Earlier studies reported a handful of core bacterial taxa consistently associated with particular populations in a few macroalgal species, including Kelp [37], *Agarophyton vermiculophyllum* [38], *Fucus vesiculosus* [28], *Ulva australis* [39] and *Ascophyllum nodosum* [40]. However, these studies were limited in scope, focusing on one location at one point in time, often without corresponding sampling of the macroalgae’ environment. Thus, outstanding questions include whether core bacteria on macroalgae are specific to host species or macroalgal clades [41] and whether they are maintained over time in multiple locations against environmental variations in bacterial communities [32].

A wide range of beneficial and detrimental interactions occur between macroalgae and their associated bacteria [42, 43]. These interactions primarily occur at the macroalgal surface, which is the physiological and ecological interface with marine bacteria and is involved in the exchange of nutrients and chemical signals [44]. The microbes involved are known only in a few striking examples, such as *Ulva mutabilis* and growth-promoting Proteobacteria (i.e., *Roseobacter*, *Sulfitobacter*, and *Halomonas*) [45]. Several bacterial taxa that directly alter macroalgal growth or development have also been identified by culture-based studies (e.g., *Pseudomonas* [46], *Rhodopseudomonas* [47] and *Pseudoalteromonas* [47]), and these readily culturable taxa that are widely found on marine surfaces [48–50]. Identifying core components of the host microbiome promises to improve our understanding of microbiome assembly and enable further investigation of bacterial roles in macroalgal hosts. The identification of macroalgae core microbes will also allow us to test the assumption that taxa that most influence macroalgal biology and physiology are part of the core or not.

Marine macroalgae are extremely diverse globally and belong to three evolutionarily distinct macroalgal clades (Rhodophyta, Chlorophyta, Phaeophyceae) that have converged ecologically [51]. Earlier studies have observed distinct epiphytic bacterial community structures across diverse macroalgal species [52]. This likely reflects the fact that differences in morphology, chemistry, and habitat result in host-species specific niches that shape bacterial communities. macroalgal species have an extensive chemical defense system against grazers and pathogens [53, 54]. For example, *Fucus* species (Phaeophyceae) produce a variety of defensive secondary metabolites, such as phlorotannins and fucoxanthin, that likely represent selective filters for the bacteria that colonize these macroalgae [55–58]. Macroalgae also exude various polysaccharides that bacteria feed upon [59], and in some cases function as antibiotics to protect the host against pathogens or fouling organisms [60, 61].

In this study, we investigate the core microbiome of a focal brown macroalga, *Fucus distichus,* by combining multiple datasets that encompass a wide range of small spatial and temporal sampling schemes. We use a simple frequency threshold as well as indicator species analysis (hereinafter referred to as “IndVal”) as a tool to capture core bacterial taxa that are constantly frequent and abundant on *F. distichus* across sites in multiple years. While the frequency method is based on data from the macroalgae alone, the IndVal method also identifies core taxa based on their enrichment compared to environmental samples (i.e. water and rock) acting as a control. We conduct these analyses at the amplicon sequence variant (ASV) level to better understand the distribution of common taxa at the finest resolution possible. We first ask (1) whether there are bacterial ASVs consistently associated with *F. distichus* across different sites and years using three approaches for the core identification, and (2) how the core ASVs defined at one place are distributed in a comparison site, 250 km away. We then ask (3) whether the core ASVs fall within macroalgal-associated clades or have been broadly characterized from other hosts and environments using broadly sampled phylogenetic trees. Finally, we ask (4) whether the core ASVs are specifically associated with *F. distichus* or are general colonizers on diverse macroalgal species by comparing to a dataset sampled from 32 sympatric macroalgal species [62].

## Methods

### Dataset description and study design

We analyzed four 16S rRNA gene amplicon datasets of the *F. distichus* microbiome, along with neighboring environmental samples from multiple intertidal locations on Calvert Island and Quadra Island, BC, Canada (Table 1). These four datasets were originally collected to address other research questions and present an opportunity to identify core bacterial taxa on *F. distichus* across multiple sites and time-points. One dataset surveyed epiphytic microbiota associated with 33 sympatric macroalgal species, including *F. distichus* on Calvert Island in March 2015 [52]. The Calvert 2018 dataset surveyed the microbiome of *F. distichus* at five sites on Calvert island in June 2018 separated by hundreds of meters to a few kilometers and found that the microbiome differed across these sites [13]. This study included common garden and transplant experiments and found that microbiome differences were stable over short time periods [13]. To avoid possible confounding effects of experimental treatments we included only samples from day 1, prior to experimental manipulation. We resampled four of these sites in June 2019 for this study to assess stability over time (Calvert 2019 dataset). The last dataset is a monthly timeseries of *F. distichus* and nearby environment from rock on Quadra Island (~ 250 km away from Calvert Island) from 2017 March through 2018 January [63]. The combined dataset is comprised of a total of 1106 samples.

**Table 1:** Overview of samples

Dataset	Data type	Sample type	North Beach (n)	Pruth bay (n)	West Beach high-tide zone (n)	West Beach low-tide zone (n)	West Beach wall (n)	Quadra (n)
2015 Calvert	Small spatial	*Fucus distichus*	0	0	**8**	**15**	0	0
		32 Other seaweeds	0	0	**38**	**208**	0	0
		Seawater	0	0	0	**27**	0	0
		Rock	0	0	**6**	**23**	0	0
2018 Calvert	Small spatial	*Fucus distichus*	**5**	**34**	**7**	**31**	**8**	0
		Seawater	0	**7**	0	**3**	0	0
		Rock	**3**	**20**	**2**	**17**	**3**	0
2019 Calvert	Small spatial	*Fucus distichus*	**5**	**5**	**5**	**4**	0	0
		Seawater	**3**	**3**	0	**3**	0	0
		Rock	**3**	**3**	**3**	**3**	0	0
2017 Quadra	Temporal	*Fucus distichus*	0	0	0	0	0	**247**
		Rock	0	0	0	0	0	**114**

Bold face indicates sample numbers included in this study

### Sample collection

Microbial DNA samples were obtained by swab from the surface of apical tip (new growth of thallus; meristem tissue) of *F. distichus.* This area was targeted for sampling because the meristem tissues of macroalgae are younger and less subjected to fouling. In kelp (a related brown alga), the bacterial communities on meristem tissue have been shown to be more consistent over time than on older blade tissues [64]. Seawater was sterilized using a 0.22 µm filter at each site and used to rinse the *F. distichus* meristematic tissue for 10 s to remove loosely associated microbes. Then this area (approximately ~ 2 cm^2^) was swabbed by rubbing a Puritan® sterile swab back and forth for 15 s. Bare rock substrates near *F. distichus* were sampled as a comparison to non-host associated microbial communities by swabbing with the same method. Adjacent seawater was filtered onto a 0.22 µm Millipore Sterivex™ unit at each sampling site to characterize microbial source pool communities; at West Beach where multiple tide heights from the same location were sampled. We used the same water for comparison to high and low intertidal sites. Swabs were stored in 2 mL sterile cryovials and Sterivex filters stored in WhirlPak™ in the field and transferred to – 80 °C freezer within 6 h until DNA extraction. Microbial DNA on sympatric macroalgal species at West beach, Calvert Island, BC was also sampled with similar methods but meristem samples could not be taken for all species; described in [52].

### Molecular methods

DNA was extracted from swabs and water filter for 16S rRNA amplicon sequencing using MoBio PowerSoil Kit (QIAGEN), following the manufacturer’s recommended protocols. PCR amplification for bacterial DNA targeted the V4 region of the 16S rRNA gene using primers, 515f: 5′–GTGYCAGCMGCCGCGGTAA–3′ and 806r: 5′–GGACTACHVGGGTWTCTAAT–3′ [65]. These primers included Illumina adapters and the forward included a 12 nucleotide Golay barcode. Then, we carried out amplicon library preparation, including PCR, quantification using Quant-IT Pico Green® ds DNA Assay Kit (Life Technologies) and pooled equal volumes (25 ng) of each sample, followed by purification using MoBio UltaClean® PCR clean-up kit. Sequencing with Illumina MiSeq using paired-end (2 × 300 bp) v3 chemistry were performed at the Integrated Microbiome Resource (IMR), Centre for Comparative Genomics and Evolutionary Bioinformatics (CGEB) at Dalhousie University according to published protocols [66].

### Bioinformatics

Raw Illumina reads were demultiplexed in pairs using the idemp tool [67] without barcode error. Sequences were merged from four datasets following quality filtering, trimming, dereplication, chimera removal, and inference of true amplicon sequence variants (ASVs). Taxonomic assignment against the SILVA 132 [68] database were processed based on exact matching between ASVs and sequenced reference strains with a standard DADA2 pipeline tutorial found at https://benjjneb.github.io/dada2/tutorial.html in R environment. In the process of filtering, we discarded ASVs if they were less than 0.1% of total number of reads or found in less than 5 samples. We also filtered ASVs with fewer than 5 reads in a sample to minimize any impact of barcode switching. Overall, we obtained 6348 amplicon sequence variants after filtering. The final products were then converted into phyloseq format in R for the downstream analysis. We then rarefied final 16S amplicon sequence products to 1500 reads per sample prior to beta-diversity analysis. We used nonmetric multidimensional scaling (NMDS) to visualize similarity among samples included in our study based on Bray–Curtis dissimilarity [69]. We also conducted permutational analysis of variance (PERMANOVA) using adonis2 by margin in the vegan package [70] in R to test for differences among bacterial communities between *F. distichus* and environmental samples. The order of variables does not matter by ‘margin’ because the significance is tested against a model that includes all other variables in the formula. All PERMANOVA statistics were generated with 9,999 permutations.

### Identifying *Fucus distichus*-core bacteria

The first method we used to identify core bacterial ASVs was a simple frequency threshold for all Calvert datasets combined. Frequency on *F. distichus* alone was calculated using the core members function within ‘microbiome’ package in R [71] with a 50% threshold. Secondly, we used indicator species (IndVal) analysis on all Calvert datasets combined to identify taxa that are both frequent on *F. distichus* and enriched on *F. distichus* compared to the nearby environment (rock and seawater). This is an extension of the frequency method. We performed IndVal analysis using multipatt function within ‘indicspecies’ package [72] in R [73]. IndVal calculates specificity (a measure of relative abundance compared to environmental rock and seawater samples) and fidelity (a measure of frequency on *F. distichus*) followed by permutation tests to evaluate the statistical significance of the bacterial association with *F. distichus* [74, 75]. We used a threshold of 0.7 IndVal value because this effectively required ASVs to be at 50% or greater frequency. The third method used was two-step IndVal analyses performed for each site and time point (a total of 11 sampling events) separately to identify indicator ASVs (those above 0.7 IndVal value). Then, the number of sampling events where an ASV was an indicator were tallied. Core ASVs were defined as those that were indicators in the majority of sites/time points, here > 6 of 11 sampling events. This two-step approach offers an alternative way to leverage multiple datasets and highlights ASVs that are consistently associated with *F. distichus*, as well as partially correcting for biases arising from unequal sample sizes across sampling events that may influence overall IndVal. Identifying indicator taxa for each site and time point also shows the variability across the dataset and allows for comparison to studies using a single site and timepoint and makes annual and regional specificity visible.

We visualized core ASVs using ggplot2 [76] in R to show their spatial and temporal distribution, frequency, and specificity compared to environmental samples. The average relative abundance and frequency of core ASVs in *F. distichus* samples was calculated from the ASV table in excel. The overall *Fucus*-specificity of each ASV was calculated by dividing a total abundance of an ASV (in *Fucus* + environmental samples) by abundance of an ASV in *F. distichus* samples.

### Phylogenetic analysis

To understand the habitat distribution of core bacteria and closely related taxa, we placed core bacterial sequences into phylogenetic trees using QIIME2 [77] with closely related sequences from the SILVA database [68] and NCBI, identified using BLAST [78]. We reformatted our data to fasta files for the QIIME2 environment using the ShortRead and seqinr package in R [79]. Sequences were aligned and the alignment masked to contain only alignment columns that are phylogenetically informative in q2-phylogeny pipeline in QIIME2 [77]. Phylogenetic tree files were then constructed by using RAxML rapid bootstrap method (replicates = 100) with GTRCAT model. We used data from the GenBank records to annotate the habitat or the identity of the host from which databased sequences were isolated. Phylogenetic tree visualization and annotation was performed in Interactive Tree of Life (iTOL) v4 [80].

## Results

### Bacterial communities of *F. distichus*

**Fig. 1:**
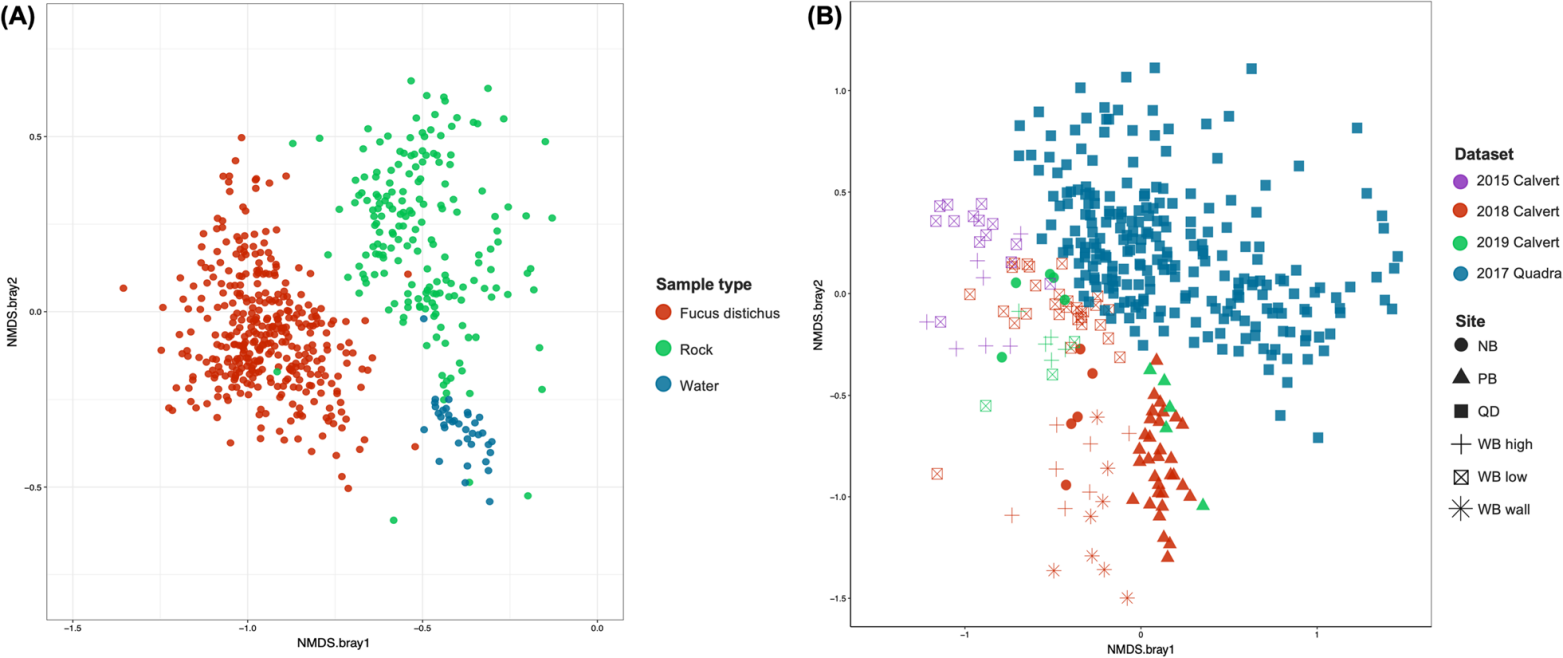
Fucus microbiota composition in comparison to neighboring environments. NMDS plots constructed from Bray–Curtis dissimilarities. **A** Microbiota on *Fucus distichus* differs from neighbouring environments (rock substrate and seawater). **B** *F. distichus* microbiota varies across sites and years

We first assessed the variability of bacterial community composition on *F. distichus* across all datasets in comparison to the environment. Bacterial communities on *F. distichus* are similar to each other across studies and distinct from the surrounding rock and seawater communities, as observed in the NMDS plot, and are significantly different from environmental samples (PERMANOVA: Pseudo-F_(2:740)_ = 66.009, R^2^ = 0.15, *p* < 0.001) (Fig. 1A). We then analyzed only *F. distichus* samples on Calvert Island and find significant differences by site and time point (PERMANOVA: Pseudo-F_(11:365)_ = 10.222, R^2^ = 0.23, *p* < 0.001) (Fig. 1B). The variability in *F. distichus* microbiome by sites and over time (a total of 11 sampling events) sets the context for identifying the core components of the microbiome. Overall, the *F. distichus* microbiome is distinct from the environment, suggesting there may be common core taxa that drive this difference. On the other hand, the variability in the *F. distichus* microbiome prompts us to look at the variation across sites and over time as well as the commonalities.

### Core bacterial taxa of *F. distichus* in Calvert datasets

We used three approaches to identify taxa that are tightly associated with *F. distichus* as candidate core taxa based on frequency alone or in combination with specificity in comparison to the environment. Throughout, our decisions are guided by the expectation that bacteria considered part of the core should be present on a majority of individuals and/or sampling events. It is important to keep in mind that the core microbiome is not an entity that exists and awaits discovery. Rather, identifying the core taxa is fundamentally a hypothesis generation tool to identify a suite of taxa that can be further tested to assess their influence on the biology of the host and the traits that underlie their commonness. Microbiome composition and variability over space and time differ across host species, and so too will the relevance of different thresholds identifying core taxa. With this framing it becomes clear that the choice of specific thresholds for defining the core microbiome are inherently arbitrary, so we present the frequency, specificity, and abundance data to allow comparison of the distribution patterns of taxa across the range of these values.

**Fig. 2:**
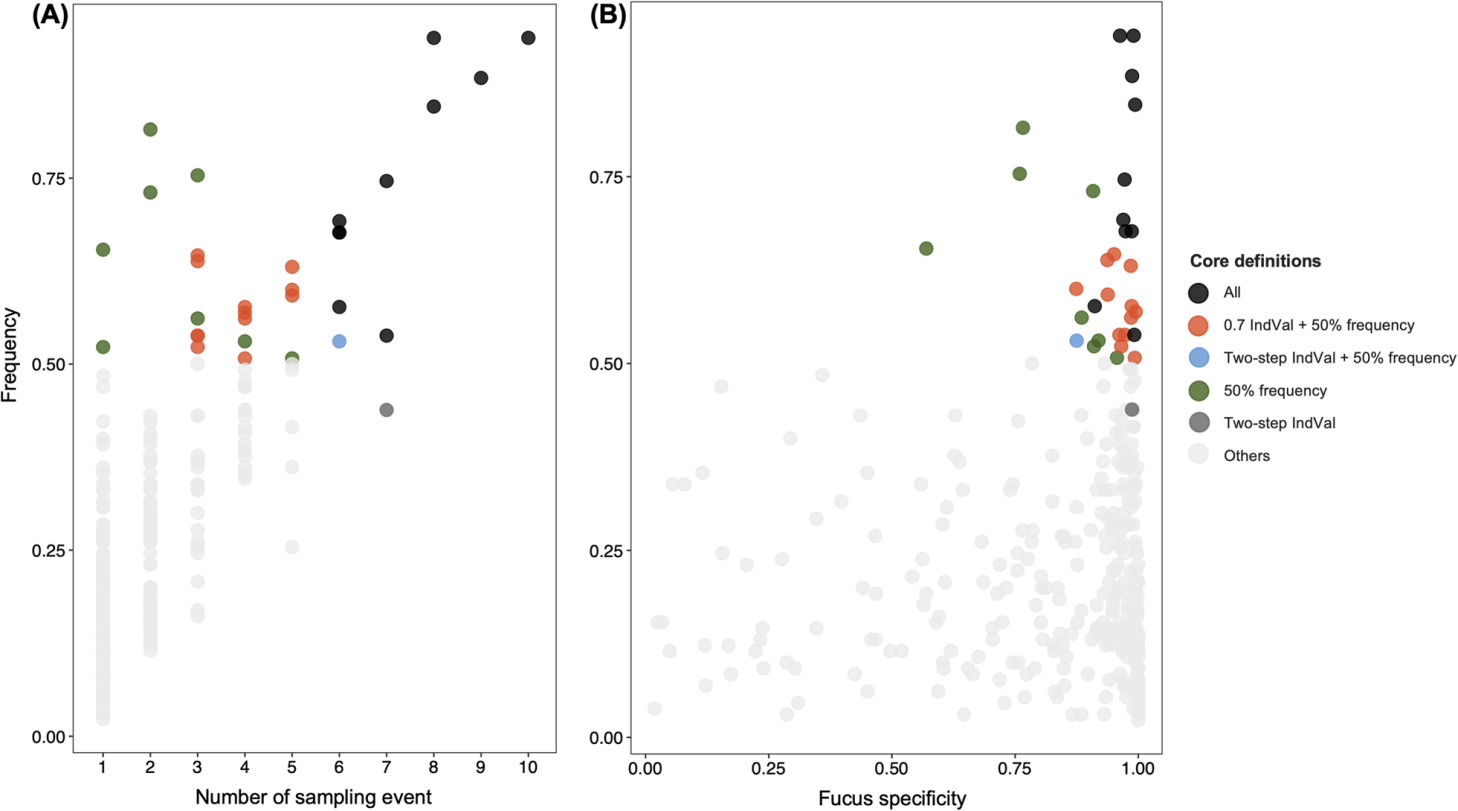
Scatter plots comparing the distribution ASVs identified as core to other *F. distichus*-indicator ASVs. Plot shows the 329 unique bacterial ASVs were detected as *Fucus*-indicators in at least one sampling event on Calvert. Frequency of bacterial ASVs across all Calvert datasets (y axis) is compared to **A** the number of sampling events in which an ASV was an indicator (0.7 IndVal) or **B** the specificity of an ASV to *Fucus* in comparison to environmental samples. Colors indicate which core definitions an ASV met

First, we used a frequency threshold method, which does not consider the occurrence of host-associated bacteria in the surrounding environment. 33 ASVs are at 50% or greater frequency across all of the Calvert Island datasets (Fig. 2), out of a total of 3,197 ASVs present in the Calvert dataset. Thus, a small portion of ASVs are found above > 50% frequency threshold within the variable *Fucus*-microbiome. We present the frequency of each ASV for ease of comparison (Additional file 2: Table S2).

Secondly, indicator species analysis (IndVal) analysis was performed over the whole Calvert Island dataset. This is an extension of frequency analysis that also incorporates specificity to *F. distichus* in comparison to the nearby environment. To define core taxa, we set > 0.7 IndVal value of a threshold. This effectively constrained *Fucus*-indicator taxa to always at greater than 50% frequency and the set of 22 ASVs identified by this approach are a subset of those identified by frequency analysis on *F. distichus* alone (Fig. 2; Additional file 1: Table S1).

Finally, we used a two-step IndVal approach to identify the bacteria that are repeatedly indicative of *F. distichus* across sites and time points. In step 1 we ran IndVal for each sampling event to identified indicator ASVs that were significantly enriched on *F. distichus* compared to the environment and frequent across individuals at a threshold of > 0.7 IndVal index value. Then in step 2 we asked which of these indicator taxa were consistent, using the criteria that an ASV had to be an indicator taxon in at least 6 of 11 sampling events. In step 1 we identified a total of 644 ASVs (329 unique ASVs) with 0.7 IndVal index threshold and found that indicator taxa are highly variable across sites and time points (Additional file 1: Table S1). In step 2, we identified 12 core bacterial ASVs (Additional file 2: Table S2). In this two-step IndVal approach, we identified a smaller set of core taxa than the other two approaches, resulting in an overlap of 11 ASVs identified by all approaches.

### Core bacteria of *F. distichus* over time

**Fig. 3:**
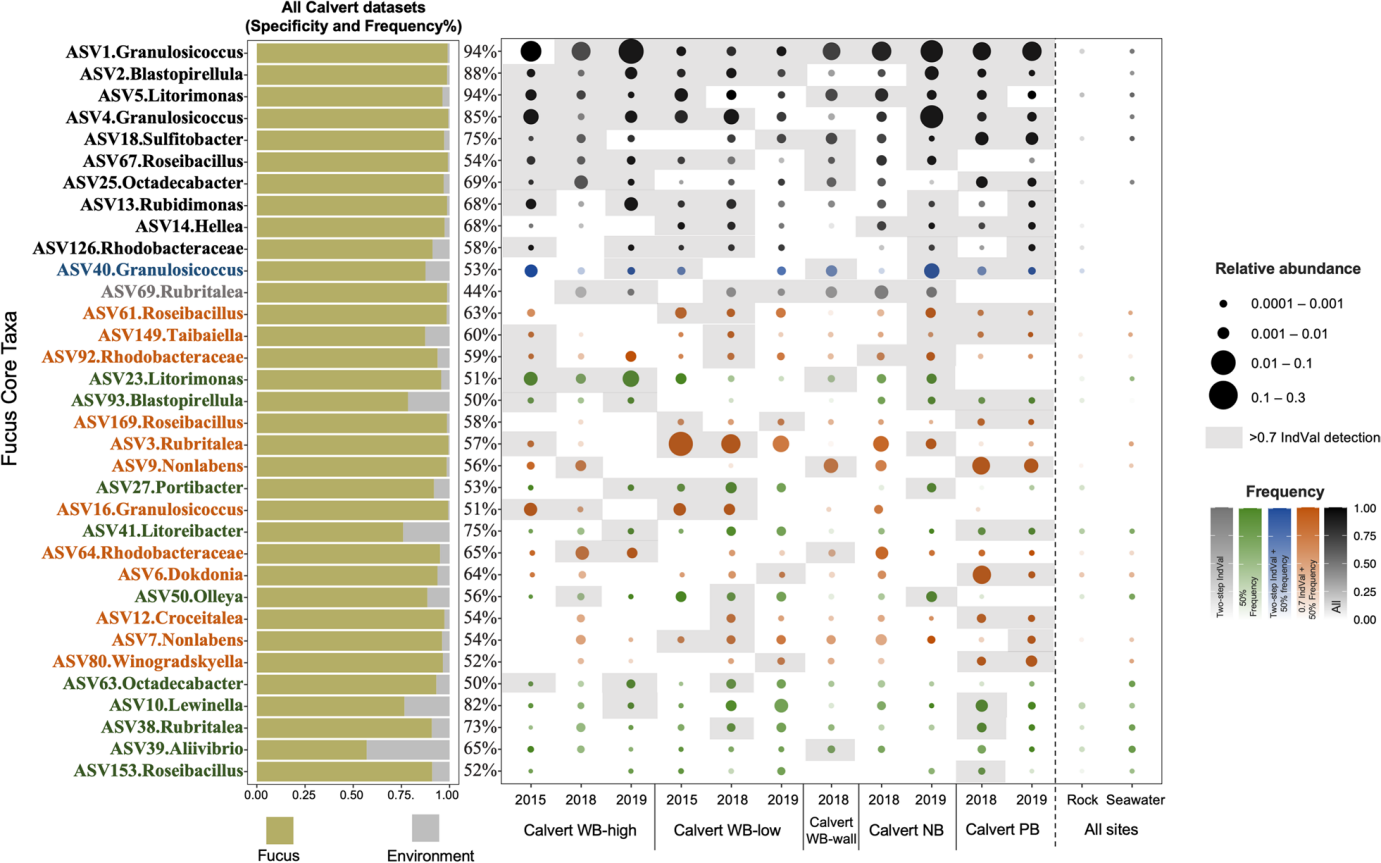
Distribution of the 34 bacterial ASVs that meet at least one core definition across *Fucus distichus* from sites around Calvert Island. The size of each dot represents average relative abundance and saturation represents frequency for each sampling event. Rock and seawater samples taken at each sampling event and are shown combined across all sampling events here. Colors indicate which core definitions and ASV met, see legend. Grey boxes indicate an ASV is an indictor of the sampling event (> 0.7 IndVal). Taxa are ordered by the number of sampling events in which they are indicators (number of grey boxes). The bar plot depicts overall specificity of an ASV to *F. distichus* (yellow bar) in comparison to the portion of sequences found on rocks and in seawater (grey bar). Frequency (percentage column) is across all Calvert datasets

We then asked whether the core bacteria identified on Calvert Island are found on a Quadra Island *F. distichus*, 250 km away. The Quadra dataset was sampled biweekly between 2017 March and 2018 January, allowing us to assess the persistence of the core ASVs over this timeseries. We used IndVal to identify the indicator ASVs from the Quadra dataset for April–June, as this was the timeframe of sampling on Calvert. We find 34 ASVs that are above the 0.7 IndVal threshold (Additional file 3: Table S3), 16 of which are also identified as core taxa on Calvert by at least one method. Interestingly, nine of the 10 ASVs identified as core by all three methods on Calvert Island *Fucus* are also indicators for Quadra *Fucus* (Fig. 3).

**Fig. 4:**
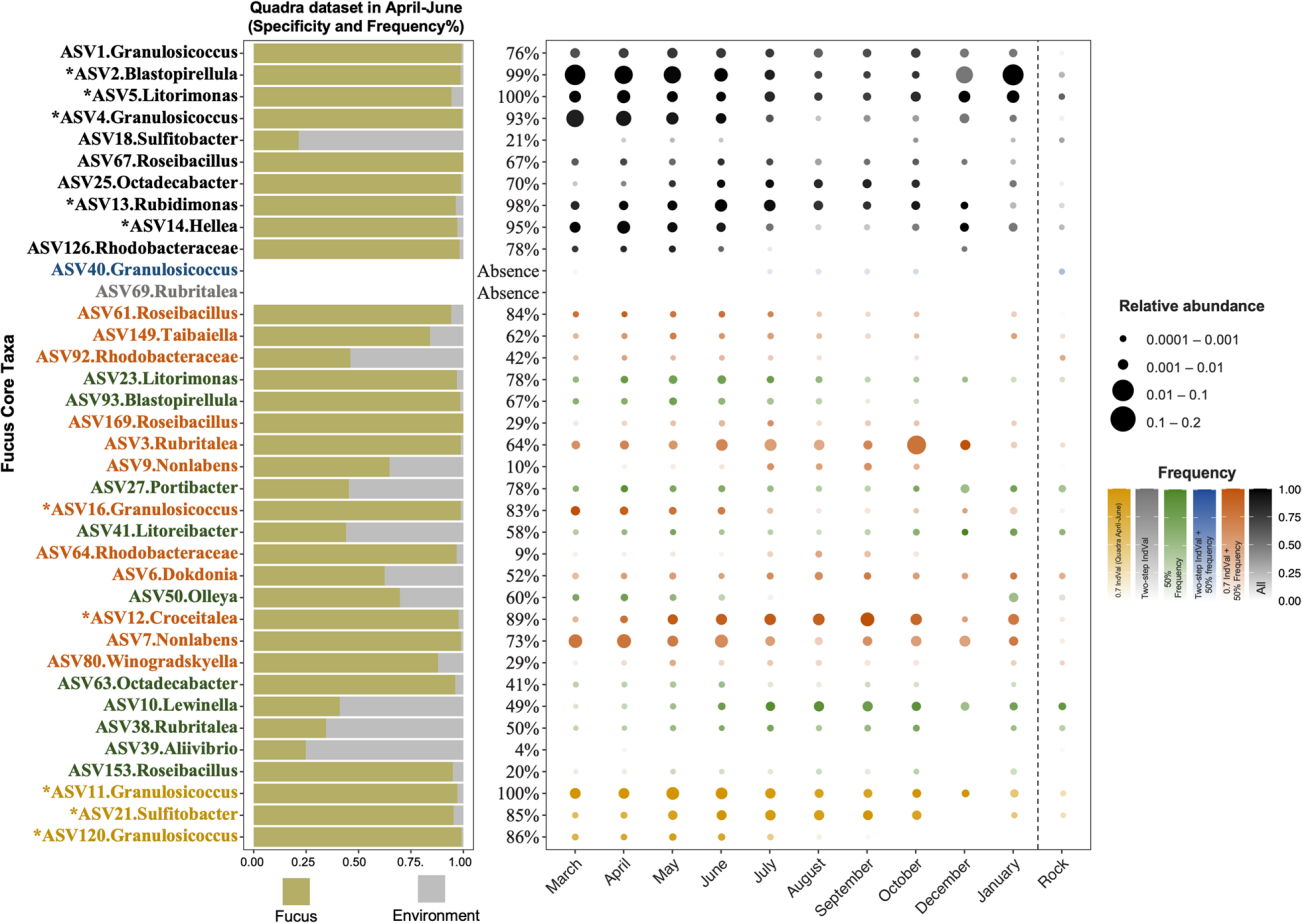
Seasonal distribution of *Fucus distichus*-core bacterial ASVs identified on Calvert in Quadra time-series dataset. *Fucus*-core ASVs determined in Calvert datasets are displayed in Quadra dataset. Quadra indicators were determined from April, May, and June samples (consistent with sampling period on Calvert) and the 10 indicators ASVs with highest IndVal scores are denoted by *, and include three ASVs that are not part of the Calvert core (in yellow); full results in Additional file 3: Table S3. Rock samples taken at each time point were combined here as an environmental comparison. The bar plot depicts specificity of an ASV to *Fucus* (yellow bar) in comparison to the portion of sequences found on rocks (grey bar), and frequency (percentage column) in Quadra dataset for April-June only. Other notes as in Fig. 2

To assess seasonality of the core ASVs, we plotted the frequency and relative abundance of the Calvert core ASVs each month. For comparison, we also plotted the distribution of the three most frequent ASVs from Quadra that are not part of the Calvert core suite (*Granulosicoccus* ASV11 and ASV120 and *Sulfitobacter* ASV21). We observe widespread seasonal variation in frequency and relative abundance, and there is a lot of variability in the seasonal patterns across ASVs (Fig. 4). For example, *Blastopirellula* ASV2 and *Granulosicoccus* ASV4 were strikingly overrepresented on *F. distichus* between March and June but decreased in relative abundance late summer to fall, whereas *Octadecabacter* ASV25 and *Croceitalea* ASV12 are more frequent and abundant July–October. Other taxa, such as whereas *Granulosicoccus* ASV1 and ASV11 and *Litorimonas* ASV5, more consistent across months (Fig. 4). Because the 16S amplicon data used in this study is compositional, it is not clear if changes in the relative abundance of core taxa are driven by decreases in absolute abundance or influenced by shifts in other taxa (Fig. 4).

### Macroalgal host specificity of *F. distichus*-core bacteria

**Fig. 5:**
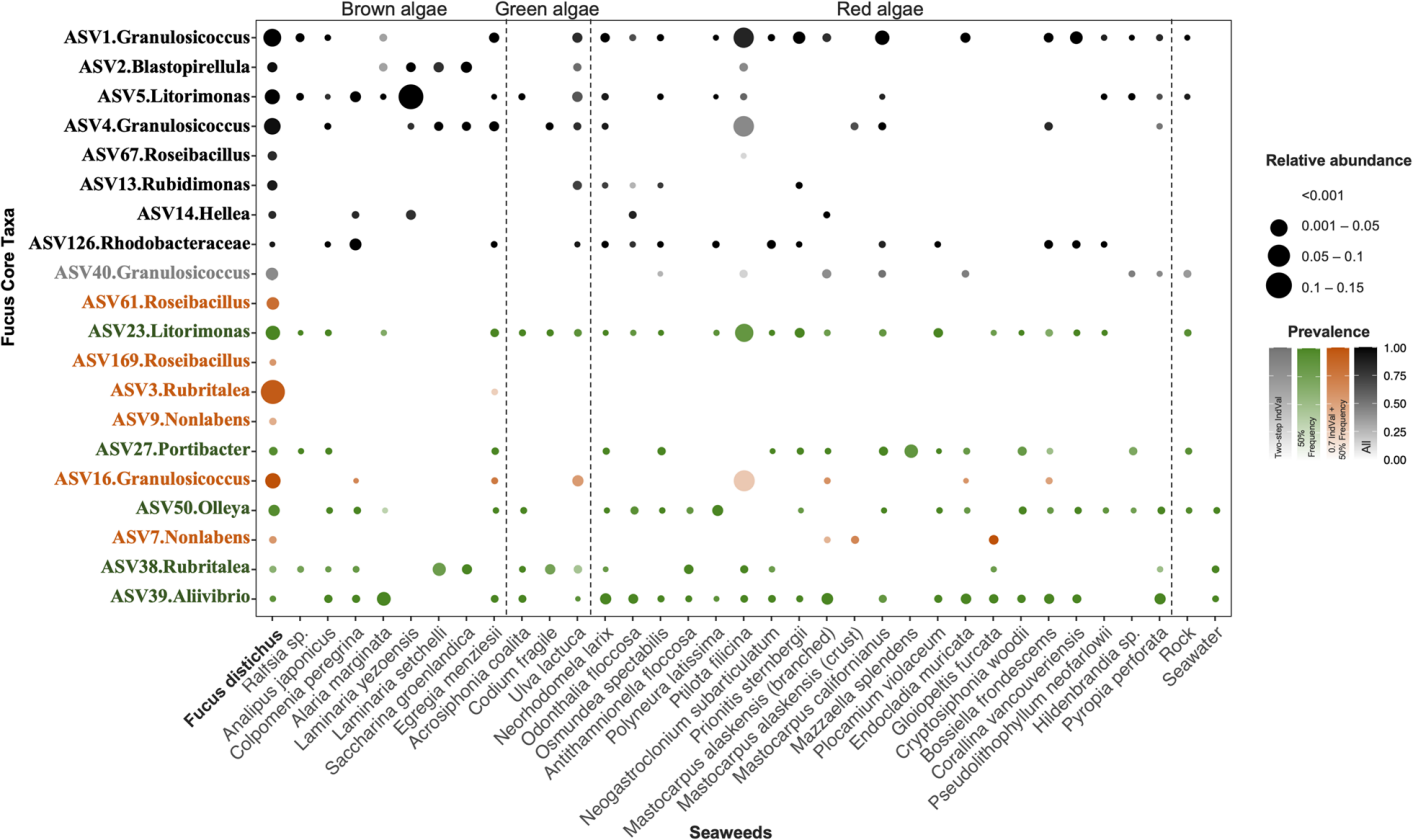
Distribution of *Fucus distichus*-core ASVs across diverse macroalgal species. The 20 *F. distichus*-core ASVs identified for Calvert (Fig. 3) that are present in the 2015 Calvert dataset (Lemay et al. 2021) are shown. Frequency and abundance are calculated from data merged across tide heights for *F. distichus* and 32 sympatric macroalgal species. Other notes as in Fig. 2

In 2015, *F. distichus* was sampled from Calvert Island alongside 32 other sympatric macroalgal species sampled at West Beach, Calvert Island, BC [52]. We used this dataset to ask whether the *F. distichus*-core ASVs were restricted to *Fucus* (*Fucus* specialists) or also associated with diverse macroalgal species (macroalgal generalists). Here we defined macroalgal generalists as bacterial taxa associated with more than one macroalgal species. We note that the threshold for presence in Fig. 5 is 0.1% average relative abundance to minimize the influence of transient taxa that have settled on nearby macroalgae. This figure also includes only the 20 core ASVs present on 2015 Calvert Island samples. Our data showed that most of *F. distichus*-core ASVs are present on other macroalgal species (Fig. 5). For instance, *Granulosicoccus* sp. (ASV1 and ASV4) and *Litorimonas* sp. (ASV5 and ASV23) were generally found on most sympatric brown, green, and red macroalgae at West Beach (Fig. 5). This comparison also reveals that a few ASVs belonging to *Rubritalea* and *Roseibacillus* (Verrucomicrobia) are found almost exclusively on *F. distichus* (Fig. 5). An IndVal analysis of *Fucus* compared to the other 32 macroalgal species showed that these taxa are significantly enriched on and highly specific to *F. distichus*; *Rubritalea* ASV3 (specificity: 0.999, *p*-value: 0.001) and *Roseibacillus* ASV61 (specificity: 0.998, *p*-value: 0.001). Of course, determining whether ASVs are *Fucus*-specialists comes with the caveat that we might not have sampled the other hosts that harbors a *Fucus*-associated ASV, while macroalgal generalists can be more easily observed when ASVs are detected on multiple macroalgal species.

### Phylogenetic placement of core bacteria

We used broadly sampled phylogenetic trees to identify the close relatives of the *F. distichus*-core ASVs. We annotated the tree with information on the isolation source from GenBank reporting the environment or host or from which sequences were isolated. This allowed us to determine whether the *F. distichus* core ASVs fall within macroalgal-associated clades or clades that have been broadly characterized from other hosts and abiotic environments. In cases where we identified multiple core ASVs belonging to the same genus, phylogenetic analysis also allowed us to determine whether the core ASVs are clustered together or fall within distinct clades. We investigated four bacterial genera among the *Fucus* core.

We found the four core ASVs within *Granulosicoccus* genus fall within distinct clades (Additional file 4: Fig. S1). The closest relatives of ASV4 were previously detected on brown macroalgae. On the other hand, close relatives of ASV1 were isolated from the environment (i.e., marine sediment and ice) (Additional file 4: Fig. S1). The close relatives of ASV16 and ASV40 did not have sequences with isolation source information were clustered with. Closely relatives of ASV5 and ASV23 within *Litorimonas* were previously isolated from brown and green macroalgae respectively (Additional file 4: Fig. S2). The closest relatives *of Blastopirellula* (Planctomycetes), core ASV2 were dominantly detected with *Fucus* species, while no sequences are clustered with ASV93 (Additional file 4: Fig. S3). Another core ASV3 within *Rubritalea* was enriched in *F. distichus* samples but nearly absent in other 32 macroalgal samples. However, we did not find any sequence that closely matched core ASV3 (Additional file 4: Fig. S4). Interestingly, *Rubritalea* ASV38 has a close relative previously detected on *Fucus vesiculosus,* but ASV38 is widespread across multiple macroalgal species (Fig. 5). Overall, the core ASVs detected in this research are all phylogenetically distinct from each other and are not artifacts. Some core ASVs have relatives that were also isolated from macroalgae, but this is not always the case.

## Discussion

Identifying core taxa has been proposed as a way to gain new insight into the complex and variable microbial communities associated with a host. However, the best approaches and thresholds to define the core are unclear. In this study, we used three approaches and an expansive dataset to identify the core bacterial ASVs associated with *Fucus distichus*. We considered 50% frequency across individuals and/or sampling events (alone or embedded in the > 0.7 threshold for IndVal) as a common sense threshold to identify core bacterial ASVs. Identifying indicator taxa at each sampling event highlights the variability in the *Fucus* microbiome (Fig. 2); there are 329 unique ASVs that are indicators in at least one sampling event (Additional file 1: Table S1). These results align with the strong differences in overall community composition previously reported for *Fucus* across sites [13] and tide heights [81] and the variable list of purported core taxa identified from one time and place [28]. Considering the common indicators across a majority of sampling events yields a much smaller set (11 ASVs) of putative core taxa and highlights the power of broad sampling.

The intersection of the three approaches is a robust suite of 10 taxa that are clearly and consistently associated with *F. distichus* (Fig. 2)*.* While we observe a clear and consistent suite of core taxa, there are no ASVs present across all *Fucus* individuals as might be expected in the case of obligate symbionts. Interestingly, 9 of the 10 core taxa identified by all methods are also part of the core for Quadra Island *Fucus*, more than 250 km away from the focal Calvert Island *Fucus* (Fig. 3). This result provides further support for the notion that core taxa identified from multiple sites and timepoints and by multiple methods are more likely to be common in other locations. Frequency analysis of the combined dataset alone or in conjunction with measuring specificity on *Fucus* compared to the environment (by IndVal) also capture these common core taxa. As expected, high frequency taxa are sometimes also common in the environment (lower specificity and excluded by IndVal). Our results suggest frequency alone captures many of the same taxa, especially when assessed across multiple sampling events, and enables using datasets that do not have environmental comparison samples. Of course, consistency does not equal functional relevance. Experimental work is needed to test the hypothesis that distribution and specificity of host-associated taxa is a good predictor of the functional influence a microbe has on the host.

We ran core microbiome analyses at the finest taxonomic resolution (ASVs) available so we could distinguish different entities within the same genus based on phylogenetic relationships and distribution. This allows us to better understand variation in host-specificity and the potential for seasonal and regional trade-offs in distribution within a genus. We find that there are multiple ASVs associated with *Fucus* within the many of the common genera that are phylogenetically distinct (Additional file 4: Figs. S1–S4) and distinct in their distribution over space (Fig. 2) and time (Fig. 3). For example, within *Granulosicoccus* there are ASVs that are consistent in relative abundance across seasons (e.g.ASV1; ASV11) and others that are rare in fall and winter (ASV4; ASV120; Fig. 3). *Granulosicoccus* also shows geographic structure: ASV11 is much more common in Quadra (100% frequency between April–June) compared to Calvert (40% frequency overall), while *Granulosicoccus* ASV1 is at 94% frequency on Calvert but at 76% frequency on Quadra. One *Litorimonas* ASV is specific to *Fucus* (ASV5), while others are common in the surrounding environment. This suggests high genetic and functional diversity within bacterial genera commonly associated with macroalgae that underlie differences in their environmental tolerance, specificity to *Fucus*, and other aspects of their functional repertoire. This variation is lumped together in studies at genus level or above.

The trade-offs in the dominant taxa and across sites and over time suggests that local population dynamics among diverse but functionally redundant ASVs could allow different microbes adapted to different abiotic conditions to fill host-associated niches across sites and seasons. Functional redundancy is common in marine microbial systems [33, 82, 83], in plants [84] and is generally a widespread pattern [83].

Host species specificity of microbial symbionts has been a long interest in many host microbiome systems [85–87], including macroalgae [88]. It is commonly suggested that a mutualist within a host ecosystem is a specialist because it has been associated with a specific lineage of symbionts over its evolutionary history [89] and a frequent microbial association (core relationship) may also be the product of evolutionary processes of host microbiomes [90]. Long-term specific associations can lead to phylogenetic congruence between symbiont and host lineages that can arise from congruent speciation for microbe and host. Recent studies found core bacterial are specialized in coral with evidence of phylogenetic congruence between symbiont and host lineages [91] and in earthworms without phylogenetic congruence. However, Host-specificity is expected to be lower for microbes that live at the interface of hosts and the free-living environment, as in the macroalgal microbiome [92].

The increasing number of studies of macroalgal microbiomes illuminates common patterns and many genera frequently associated with a diverse array of macroalgal species. For example, the most common *F. distichus*-associated core genera in this study were *Granulosicoccus* and *Litorimonas*, which are also dominantly found in the broad range of brown, green and red macroalgal species: *Fucus* species [93, 94], *Nereocystis luetkeana* [95], *Laminaria setchellii* [62], *Macrocystis pyrifera* [35], *Ecklonia cava* [96], *Caulerpa cylindracea* [97], *Ulva rigida* [98], *Porphyra umbilicalis* [99] and *Gelidium lingulatum* [100]. Are these generalists or are there specialized lineages within each genus that specifically associate with each species? We analyzed the distribution of *F. distichus* core ASVs across 36 other macroalgal species to answer this question. We find that *F. distichus* core ASVs are generalists: most are also associated with a broad range of other macroalgae (Fig. 5). There are multiple distinct ASV lineages within most of the common genera that are part of the core (Fig. 3). A few potential specialists in the Verrucomicrobia (*Roseibacillus* ASV61, ASV67 and *Rubritalea* ASV3) also emerged in our comparison of 32 sympatric macroalgae (Fig. 5). Interestingly, these are not found at high frequency in the overall *Fucus* microbiome datasets. Thus, the expectation that more tightly associated bacteria are more likely to be host specific is not supported. Overall, our research suggests the generality at the ASV level is common and co-evolution at the host species level is very unlikely to be occurring in macroalgae.

The large number of core taxa identified for *F. distichus* and high degree of variation in the indicator taxa are potentially related to the observation that most of the *F. distichus*-core are macroalgal generalists. These patterns suggest that *Fucus*:microbe associations are flexible and likely arise because bacteria that colonize *Fucus* are responding to signals that are widespread, and/or that the mechanisms that promote *Fucus*:microbe association are general in nature [92]. Specific marine bacterial strains may colonize on the surface of diverse macroalgae for the degradation of the polysaccharides from macroalgae [101, 102]. For example, major cell wall polysaccharides are widely distributed across green, red and brown macroalgae [103, 104]. Dimethylsulfoniopropionate (DMSP) is another widely produced molecule that marine bacteria have been shown to respond to and it is produced by a wide array by marine algae. [105–107]. Bacterial symbionts of the green macroalga *Ulva* attracted by DMSP and appear to use it as a reliable indicator of a food source, in this case glycerol [108]. DMSP can also be used directly as a food source by some bacteria including *Granulosicoccus* and *Litorimonas* [109, 110]. The ability to recognize and metabolize DMSP could promote their ability to colonize diverse macroalgal species, and may underly bacteria:macroalgae associations in other cases.

Next research steps should include directly testing the prevailing hypothesis that core bacteria are more likely to play a functional role in host biology [15]. Though culturing marine bacteria remains a major challenge, recent efforts using different types of media enabled the culture of a remarkably high portion of the bacteria associated with brown macroalga, *Ectocarpus* [111] and put direct, culture-based tests within the realm of possibility. Such studies should be carefully designed and adequately test the alternative possibility that many marine and biofilm-associated bacteria influence macroalgal biology. Earlier studies showed bacterial strains that are readily culturable are commonly among the strains with growth promoting effects [112, 113] or defense against macroalgal pathogens [114, 115].

## Conclusion

We identified a suite of core taxa that are consistently associated with the macroalgae *Fucus distichus* using three alternative approaches. We expect these methods will be useful in other systems for identifying core taxa when different types of data are available. Visualizing the distribution of frequency and specificity are likely to aid in identifying appropriate thresholds across a range of host:microbe systems that differ in their biology and the fidelity of microbial interactions, and will be helpful in uncovering ASVs with distributions that are restricted by region, over time, or to particular hosts. We argue that broader sampling schemes are more likely to yield a robust suite of core microbes overall and identify the distribution patterns of taxa restricted in place and time. These data can be used to inform robust experimental tests of the hypothesis that the core microbiomes are more likely to be biologically important symbionts.

## Supplementary Information

**Additional file 1**. Supplementary Table 1 showing indicator ASVs of *F. distichus* on Calvert Island for each sampling event in the two-step IndVal.

**Additional file 2**. Supplementary Table 2 showing overlapping indicator ASVs of *F. distichus *on Calvert Island across sampling events and their overall specificity to determine core taxa. In step2, *F. distichus*-indicator ASVs at greater than 0.7 IndVal index are evaluated. 12 bacterial ASVs determined by the two-step approach are in bold.

**Additional file 3**. Supplementary Table 3 showing indicator ASVs of *F. distichus* in the time-series Quadra dataset, including April, May and June samples.

**Additional file 4**. Maximum likelihood phylogenetic trees of core bacterial taxa within *Granulosicoccus*, *Litorimonas*, *Blastopirellula* and *Rubritalea*.

## Data Availability

All datasets within phyloseq format and bioinformatic scripts from this study are available at (https://github.com/Jungsooparkubc/Fucus-Core-Microbiome). Sequences and metadata are deposited in the European Nucleotide Archive under the project accession PRJEB57342. All relevant data for Indicator Species Analysis are within the main paper and its Additional files 1, 2, 3 and 4.

## Abbreviations

ASV: Amplicon sequence variants
IndVal: Indicator species analysis
DMSP: Dimethylsulfoniopropionate
NMDS: Non-metric multidimensional scaling
PERMANOVA: Permutational analysis of variance
NB: North Beach site on Calvert Island
PB: Pruth Bay site on Calvert Island
WB-L: West Beach at low tide on Calvert Island
WB-H: West Beach at high tide on Calvert Island
