# Supplementary material for: Alternative approaches to identify core bacteria in Fucus distichus microbiome and assess their distribution and host-specificity
Source: Environ Microbiome. 2022 Nov 16;17:55. doi: 10.1186/s40793-022-00451-z (PMC9670562; doi:10.1186/s40793-022-00451-z)
Supplement: Supplementary file 4 — Additional file 4. Maximum likelihood phylogenetic trees of core bacterial taxa within Granulosicoccus, Litorimonas, Blastopirellula and Rubritalea. [file 40793_2022_451_MOESM4_ESM.docx]

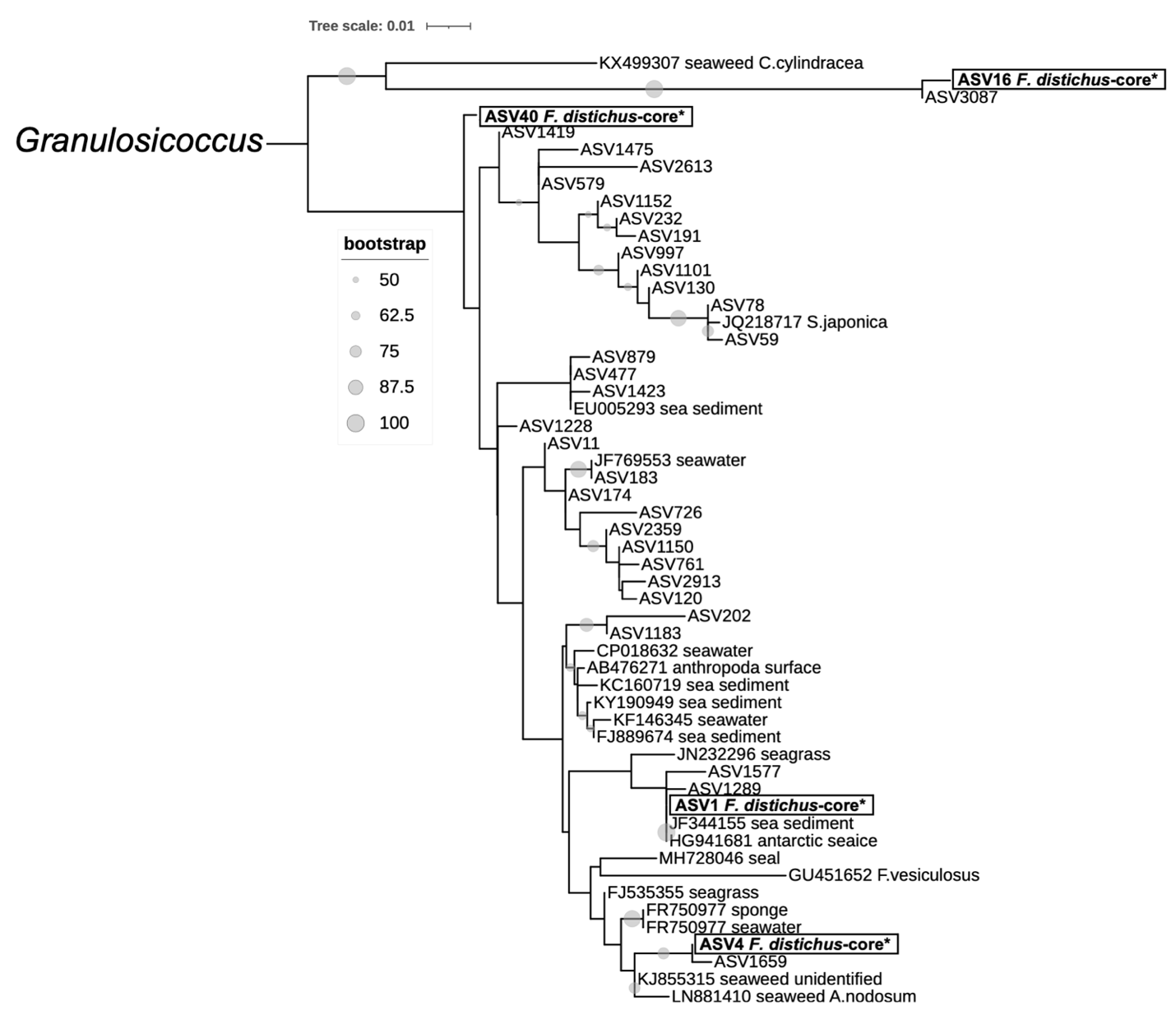


**Supplementary Figure.1 | Maximum likelihood phylogenetic tree of *F. distichus*-core taxa within *Granulosicoccus* clade constructed in RAxML with rapid bootstrapping.** Sequences from this study are labeled by ASV number and core ASVs are denoted with an * and placed within a box. Database sequences are labeled with GenBank accession number followed by reported isolation habitat or host. Bootstrap values are denoted by dot size on branches.

**
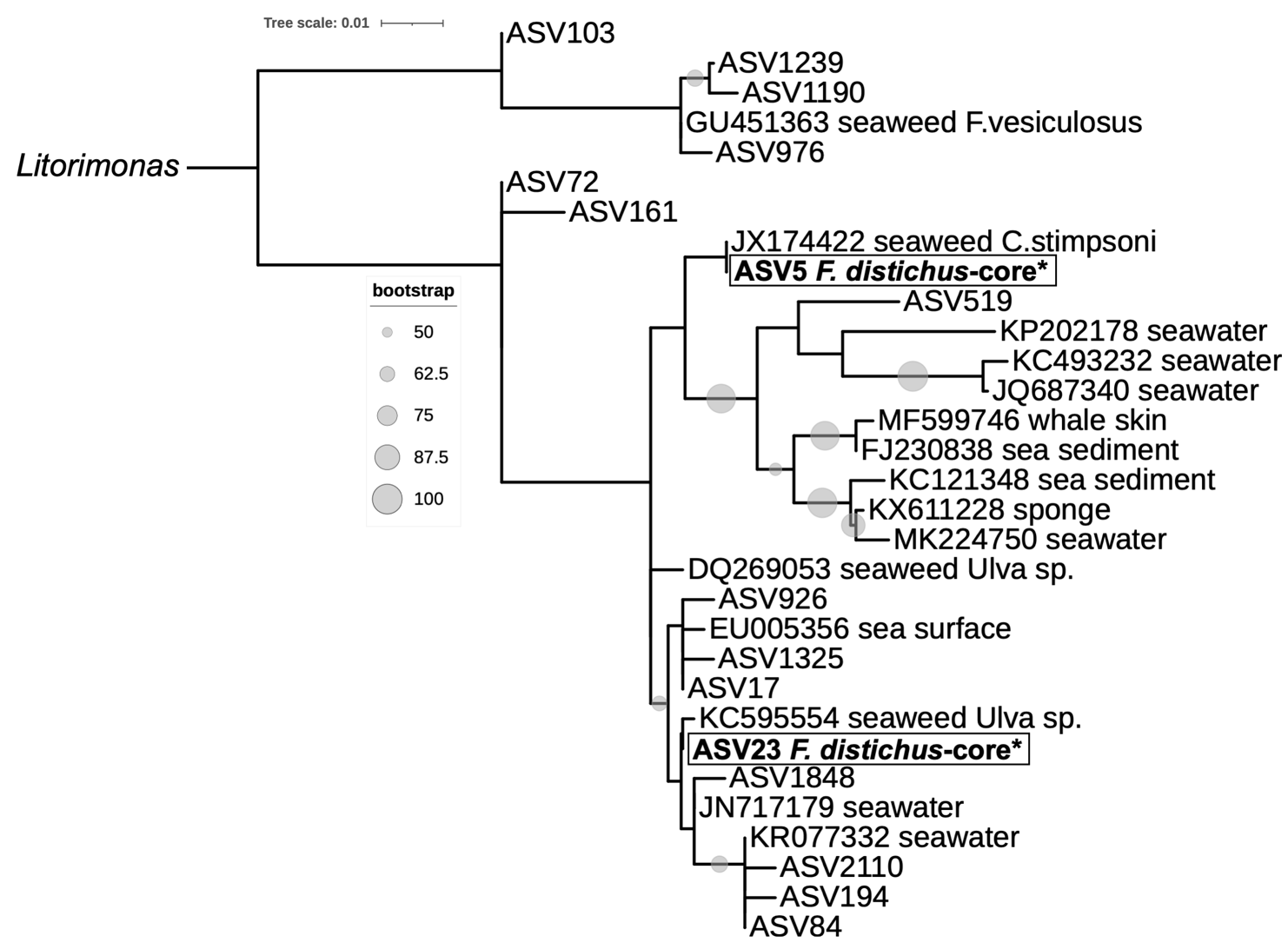
**

**Supplementary Figure.2 | Maximum likelihood phylogenetic tree of *F. distichus*-core taxa within *Litorimonas* clade constructed in RAxML with rapid bootstrapping.** Other notes as in Figure S1.


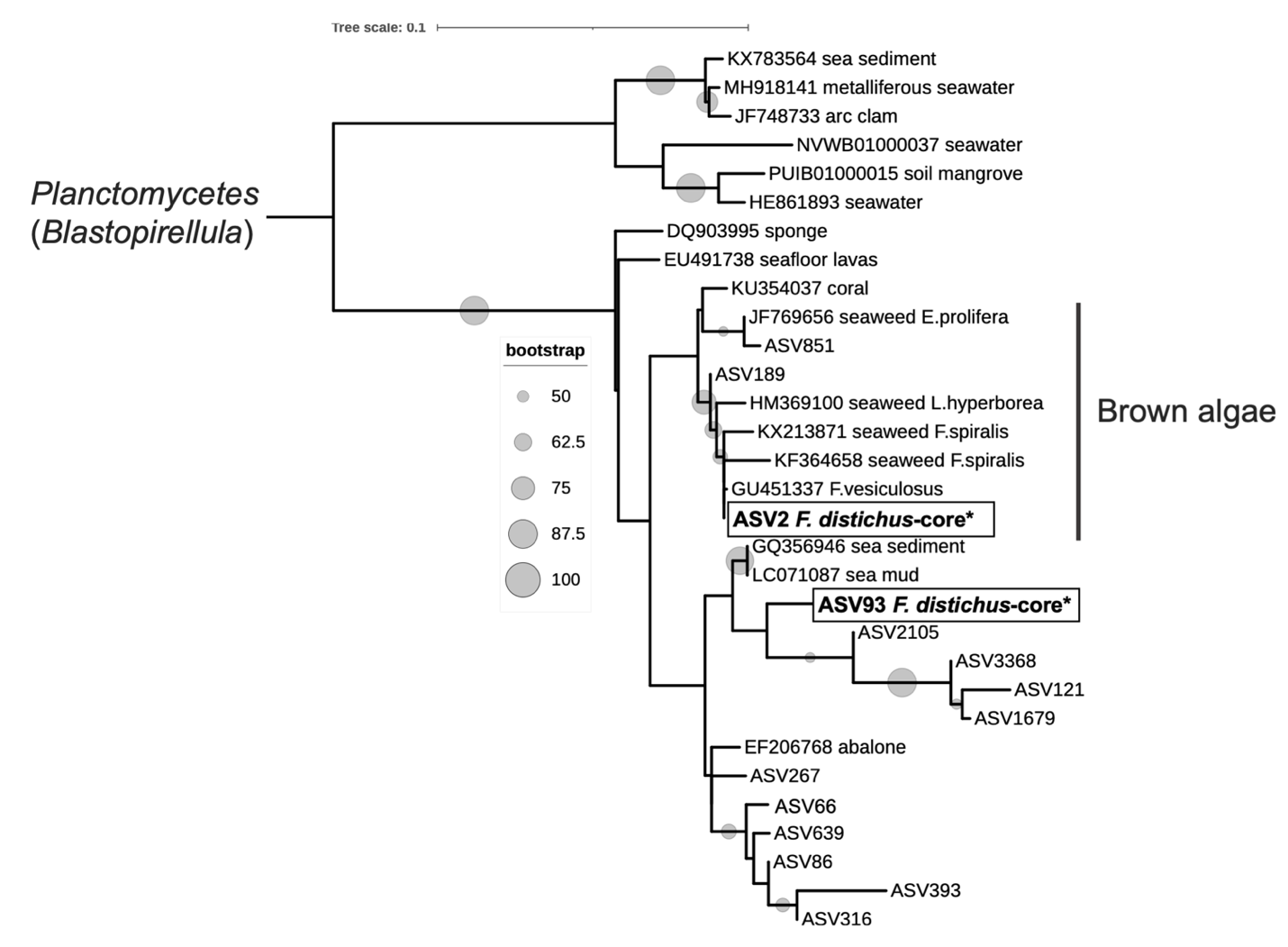


**Supplementary Figure.3 | Maximum likelihood phylogenetic tree of *F. distichus*-core taxa within *Blastopirellula* clade constructed in RAxML with rapid bootstrapping.** Other notes as in Figure S1.


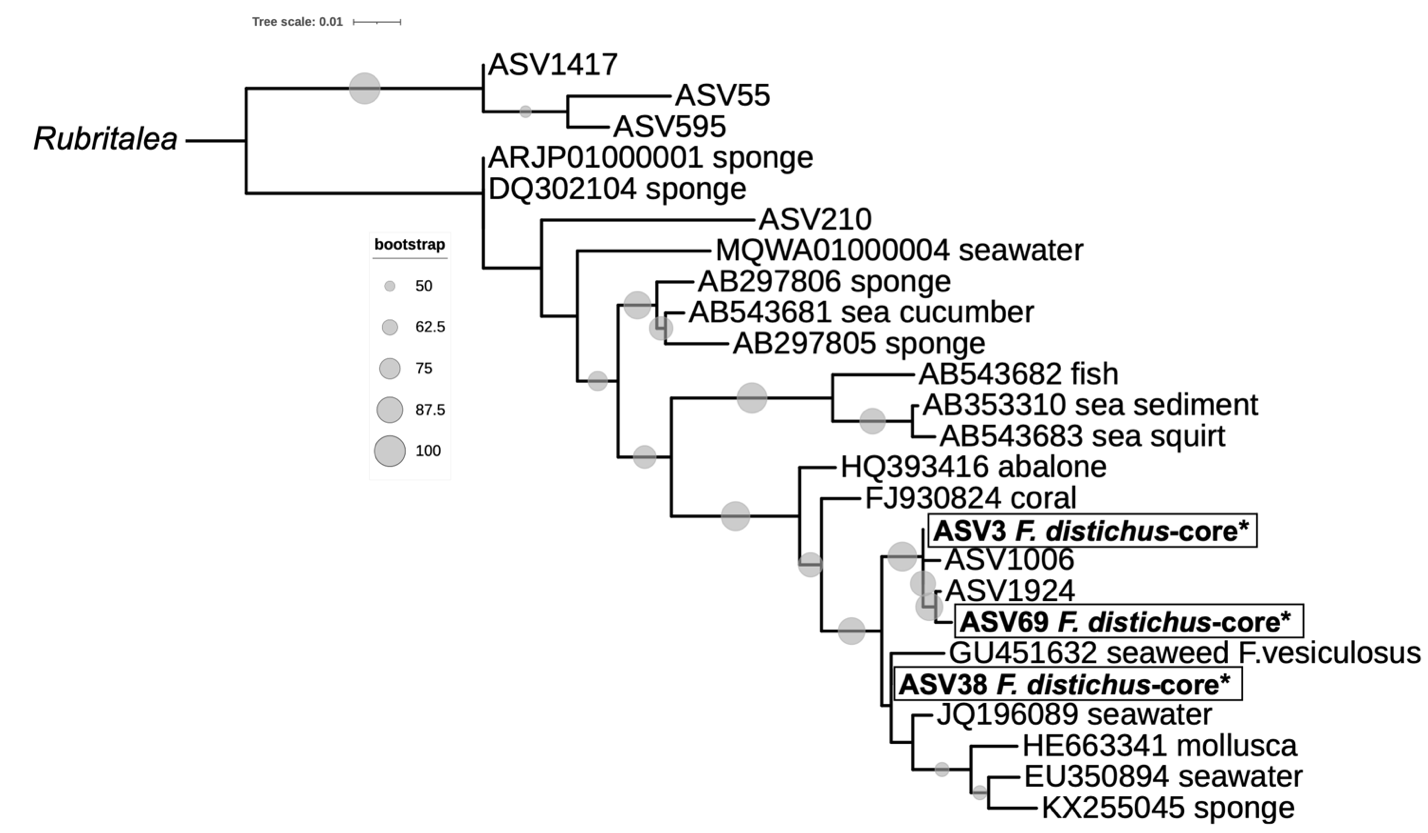


**Supplementary Figure.4 | Maximum likelihood phylogenetic tree of *F. distichus*-core taxa within *Rubritalea* clade constructed in RAxML with rapid bootstrapping.** Other notes as in Figure S1.
